# Liquid-liquid phase separation: an emerging perspective on the tumorigenesis, progression, and treatment of tumors

**DOI:** 10.3389/fimmu.2025.1604015

**Published:** 2025-06-26

**Authors:** Qin Jian, Qi Xu, Sirui Xiang, Rongrong Wang, Chuchu Wang, Boxun Zhang, Ruli Li, Junzhi Lin, Chuan Zheng

**Affiliations:** ^1^ School of Clinical Medicine, Chengdu University of Traditional Chinese Medicine, Chengdu, China; ^2^ TCM Regulating Metabolic Diseases Key Laboratory of Sichuan Province, Hospital of Chengdu University of Traditional Chinese Medicine, Chengdu, China; ^3^ College of Basic Medicine, Chengdu University of Traditional Chinese Medicine, Chengdu, China; ^4^ Department of Endocrinology, Hospital of Chengdu University of Traditional Chinese Medicine, Chengdu, China; ^5^ Sichuan Provincial Engineering Research Center of Innovative Re-development of Famous Classical Formulas, Tianfu TCM Innovation Harbour, Chengdu University of Traditional Chinese Medicine, Chengdu, China

**Keywords:** liquid-liquid phase separation (LLPS), tumor, emerging perspective, tumorigenesis, progression, treatment

## Abstract

Research in the field of Liquid-liquid phase separation (LLPS) breaks through the classical theory of gene mutation in the mechanism of tumorigenesis and provides a new perspective for comprehending tumors from a network regulation standpoint. Although there have been some reviews discussing the relationship between LLPS and tumors, they often focus on elaborating isolated mechanisms. In the face of complex and diverse disease characteristics, it is necessary to summarize the correlation between LLPS and tumors through a linked and holistic approach to reveal the deep-rooted relationships among tumor disease mechanisms. Therefore, we adopt a dual-dimensional analytical framework, where one dimension (the longitude) integrates cellular physiology, tumorigenesis, progression, and therapeutic responses, while the other dimension (the latitude) focuses on the pathogenic characteristics of tumors. This structural design enables comprehensive analysis of LLPS functions across both dynamic processes and pathological features. This article first outlines how LLPS regulates normal cellular physiological activities, such as gene expression, DNA damage response (DDR), and epigenetic modifications. It then summarizes how LLPS malfunction promotes tumorigenesis and progression, including the oncogenic processes of fusion oncoproteins (FOs) expression, tumor suppressor gene mutation, epigenetic modification defect, and DDR repair abnormality, as well as the tumor progression processes of proliferation and metastasis, dysregulation of autophagy, and metabolic reprogramming. Promising therapeutic strategies are then proposed. Finally, the existing research is prospected. The above insights drive the innovation of LLPS-based tumor therapeutic strategies and the development of targeted antitumor drugs.

## Graphical Abstract

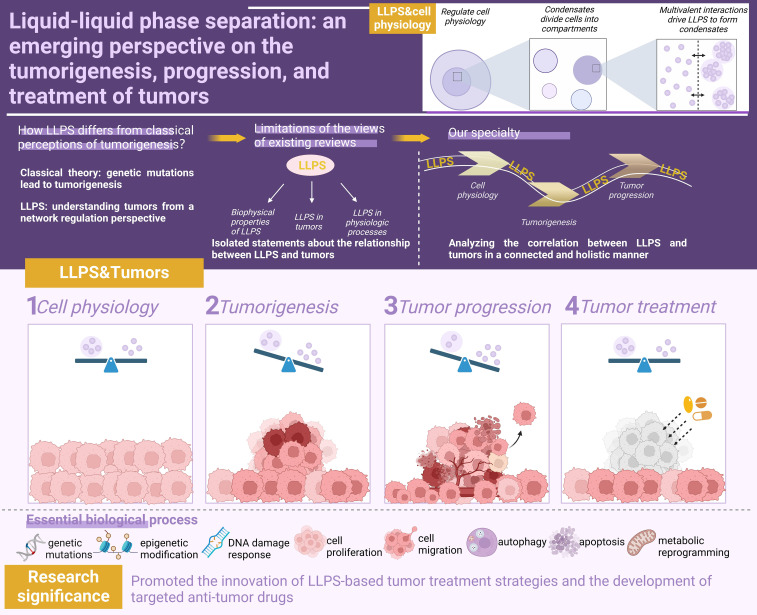

## 1 Introduction

More than a century ago, scientists discovered the presence of membrane-free compartment-like structures in cells (1), but they did not understand the specific functions of these structures in cells. In 2009, Brangwynne et al. (2)introduced the phase transition concept into biology for the first time by discovering that the membraneless P granules of Caenorhabditis elegans have liquid-like characteristics (capable of rapidly dissolve and condense), which coincides with the phase transition concept in physics. This is of epoch-making significance for the study of the biophysical properties of membrane-free structures in cells, and the specific functions carried by these properties are important areas for scientists to further explore in depth. In 2011, the team further revealed the critical role of phase transition in organelle assembly and found it to be a central mechanism for nucleolus formation (3). The discovery of this important function has spurred extensive research by scientists, who have adopted the term ‘Liquid-Liquid Phase Separation (LLPS)’ to more accurately describe how biomolecules maintain the normal physiological activities of cells (4).

LLPS refers to the phenomenon whereby biomolecules condense and separate from the surrounding liquid to form droplet-like structures (also known as biomolecular condensates) when the concentration of biomolecules exceeds a certain threshold (5, 6). Intrinsically Disordered Regions (IDRs) serve as the structural foundation for LLPS. Their amino acid sequences regulate the LLPS process through dual mechanisms: (1) by establishing multivalent interaction binding sites and chemical properties (such as hydrophobicity, electrostatics interactions, and polarity) to mediate the process (7), and (2) by leveraging conformational dynamics characteristics to influence LLPS behavior (8). Notably, aromatic amino acids—traditionally regarded as key stabilizers of protein three-dimensional structures (e.g., promoting globular protein folding) (1, 9)—are typically underrepresented in IDRs due to their lack of fixed structure. However, recent studies reveal that aromatic amino acids retained within IDRs are critical for LLPS. These residues provide essential and reversible molecular interactions—via weak π-π or cation-π interactions—to facilitate the formation of dynamic biomolecular condensates (10).

Biomolecules drive LLPS through multivalent interactions to form compartments that are compartmentalized from each other, thereby precisely regulating cells in both temporal and spatial dimensions, which not only supports basic cellular activities but also promotes the diversity and specificity of cellular functions, which is important for cellular adaptations to environmental changes, maintenance of homeostasis, and execution of complex physiological functions (11). Dysfunctions in this mechanism lead to disturbances in cellular function, and thus LLPS malfunction is a key event in the onset and/or progression of a wide range of diseases (12). More and more research confirm that biomolecular condensates are key regulators of many cancer cell pathologic processes (13), such as lung cancer (14, 15), breast cancer (16), colorectal cancer (17), and leukemia (18). Targeted therapies against LLPS and/or biomolecular condensates are expected to be a new strategy in the fight against cancer. For example, disrupting EML4-ALK LLPS and impairing STAT3 phosphorylation may be a novel treatment for EML4-ALK-positive lung cancer (15). In addition, LLPS has been associated with the mechanism of tumor drug resistance generation, and LLPS-based tumor drug resistance studies have provided novel insights into understanding and solving the challenges of radiotherapy and chemotherapy resistance. For example, inhibition of the LLPS process that induces tumor chemoresistance enhances therapeutic sensitivity (19).

**Figure 1:**
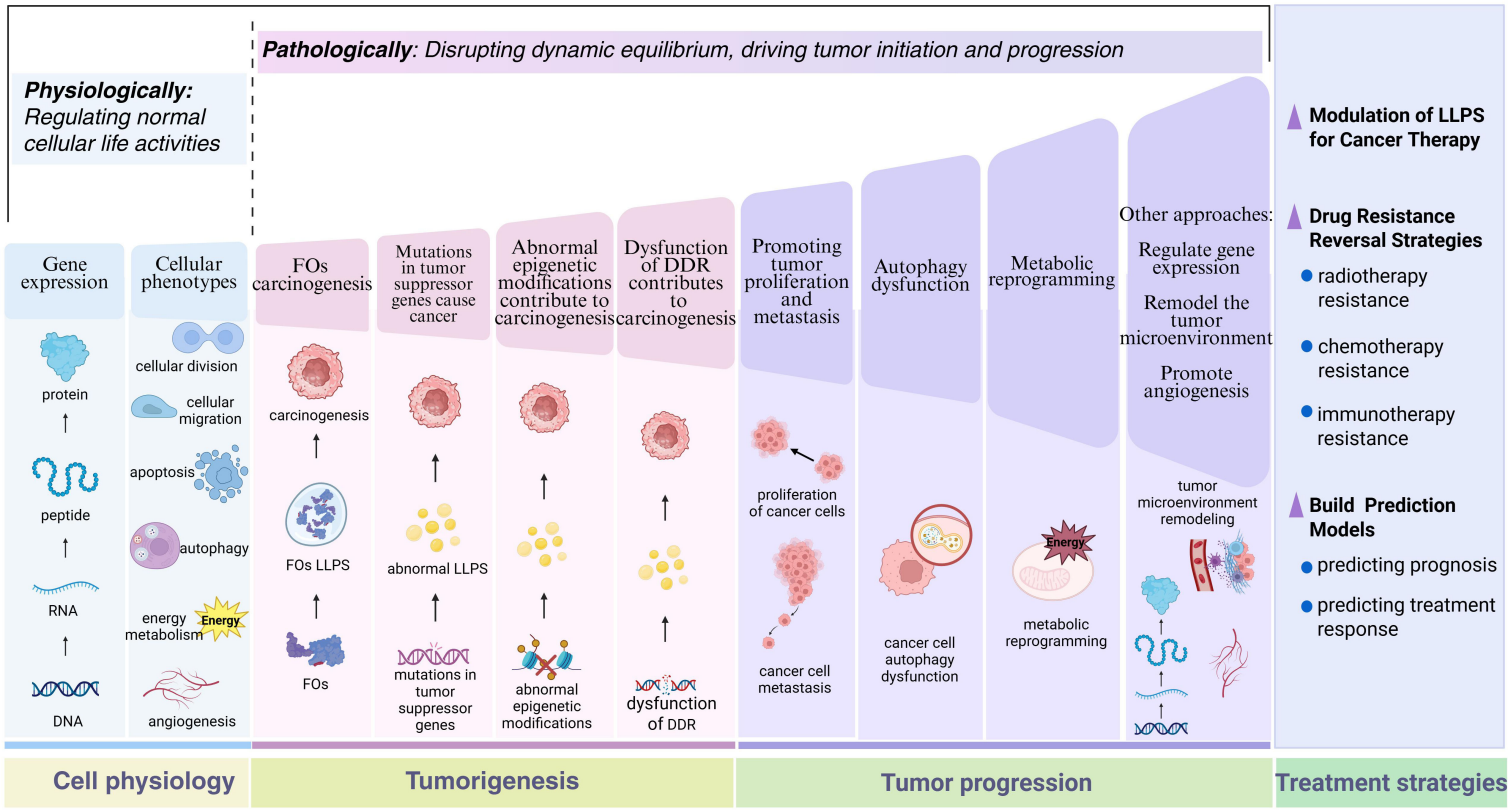
The role of LLPS in normal cellular physiology and tumor-associated processes. This schematic systematically elucidates the biological functions of LLPS from the perspective of pathological evolution. In normal cells, LLPS dynamically maintains cellular homeostasis, including precise regulation of gene expression networks and cellular phenotypes. During carcinogenesis, LLPS exerts effects in diverse oncogenic pathways, such as FOs, mutated tumor suppressor genes, abnormal epigenetic modifications, and dysregulated DDR. In advanced tumor progression, LLPS regulates multidimensional malignant phenotypes, involving enhanced proliferation/metastasis potential, autophagy dysfunction, and metabolic reprogramming. Therapeutic strategies based on LLPS mechanisms achieve breakthroughs, encompassing the development of LLPS-targeting inhibitors, novel approaches for reversing drug resistance, and construction of prognostic evaluation models. By contrasting the functional heterogeneity of LLPS under normal and pathological states, this figure not only reveals the dual nature of phase separation regulation but also provides a visual paradigm for establishing an integrated cognitive framework spanning from basic mechanisms to clinical translation. Figure created in BioRender. (2025) https://BioRender.com/5lasov6.

To thoroughly elucidate the central role of LLPS in tumor biology and provide theoretical support for its clinical therapeutic applications, there is an urgent need to systematically integrate cutting-edge research findings on LLPS in cellular homeostasis maintenance, tumor evolution processes, and therapeutic responses. **Figure 1** visually illustrates the dynamic mechanisms by which LLPS regulates cellular fate under normal physiological and pathological tumor conditions. By comparing functional transitions of LLPS between normal and diseased states, this figure systematically reveals its diverse functions, offering a framework perspective for understanding its complex biological roles.

## 2 LLPS is an important way to regulate cell physiology

Classical intracellular membrane-bound organelles, including the Golgi apparatus and mitochondria, are surrounded by phospholipid bilayer membranes that form separate compartments and perform life activities such as signaling, autophagy, and cellular stress responses through membrane contact sites (20, 21). In addition to these membrane structures, various membrane-free structures formed by biomolecules via LLPS exist in cells (1), such as nucleoli, Cajal bodies, stress granules, and P-bodies (22), which play important roles in the transmission of genetic information, stability of the internal environment, and other processes (20). The above studies illustrate that LLPS has become a driver for the formation of important cellular structures. Notably, LLPS is not only involved in the formation of the membrane-free structures described above, but also plays a critical role in regulating cellular physiological processes in a broader context, especially in regulating gene expression patterns and influencing the overall cellular phenotype.

### 2.1 Patterns of gene expression regulation involving LLPS

Stable transmission of genetic material involves key processes such as gene expression, epigenetic modification and DNA damage response (DDR). LLPS-mediated regulatory mechanisms effectively maintain the stability of genetic material by affecting transcription factor activity, altering chromatin structure and promoting DNA repair.

#### 2.1.1 Protein synthesis-related processes involving LLPS

**Figure 2:**
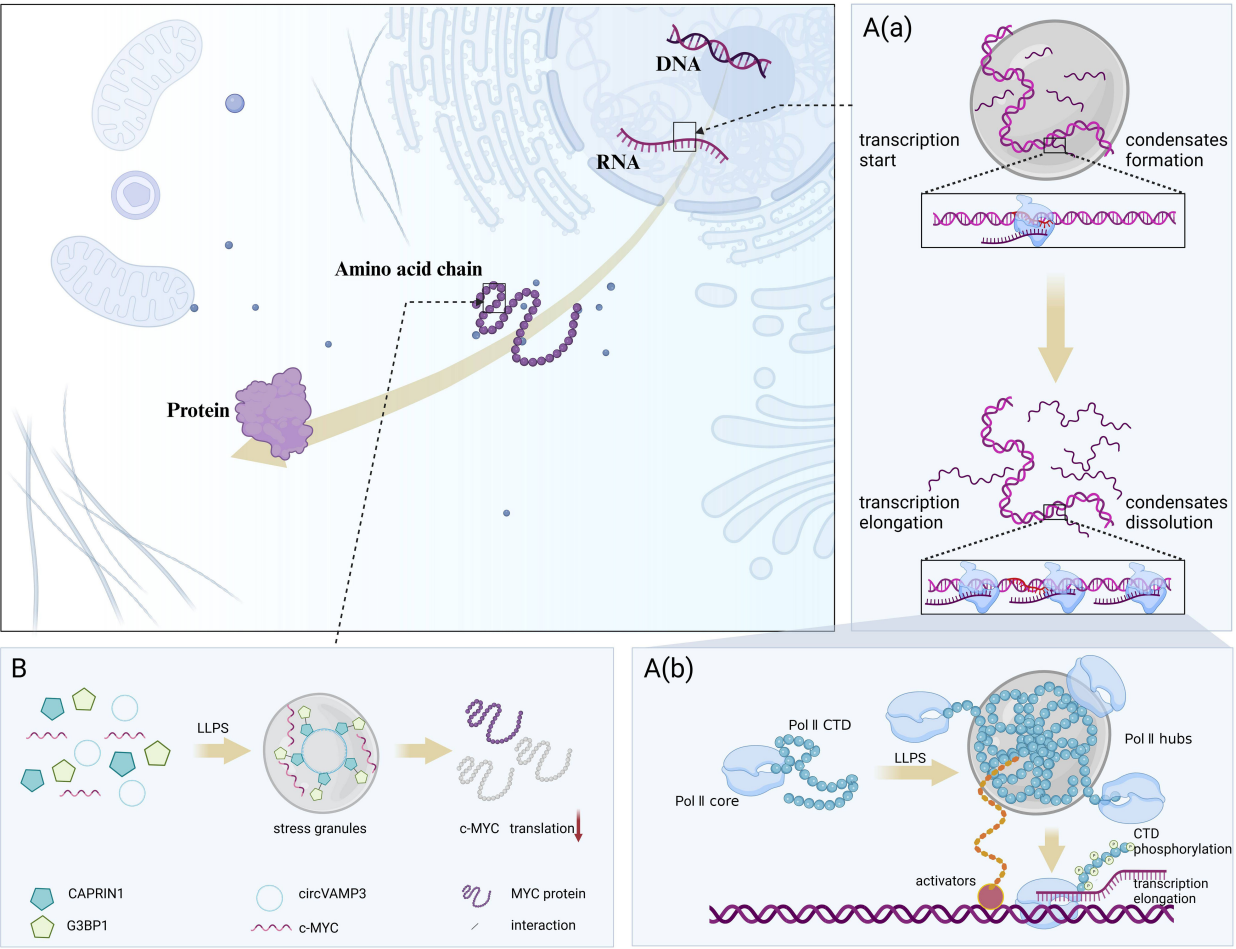
LLPS regulates protein synthesis-related processes. LLPS dynamically coordinates the on-off switching of transcription and translation by responding to RNA concentrations and environmental changes. **(A)** LLPS involved in the feedback mechanism of transcriptional regulation. **(A,** a**)**. In the early stages of the transcription process, low levels of RNA promote the formation of transcriptional condensates, whereas during transcription elongation, high levels of RNA contribute to condensate dissolution. **(A,** b**)**. RNA Pol II forms hubs on active genes through interactions between CTD and interactions with activators, and releases RNA Pol II from the hubs through CTD phosphorylation for promoter escape and transcription elongation. **(B)** LLPS involved in the translation. circVAMP3 interacts with CAPRIN1 under stress conditions to drive LLPS to form stress granules and inhibit proto-oncogene c-MYC translation. Figure created in BioRender. (2025) https://BioRender.com/w54d237.

The activation domains of some transcription factors (TFs) consist of IDRs that bind to mediator complexes in various conformations, which in turn undergo LLPS driven by multivalent interactions to form condensates that favor transcriptional activation (23). Based on this principle, scientists have developed a gene activation system called DropCRISPRa, in which the FETIDR-AD fusion protein forms phase-separated condensates at specific genomic sites, efficiently aggregating endogenous BRD4 proteins and RNA Pol II containing S2 phosphorylated C-terminal domains, thereby facilitating the transcription elongation process, and the critical role of LLPS in efficient gene activation was experimentally verified (24). In addition, LLPS is involved in RNA-mediated transcriptional regulatory feedback mechanisms, i.e., in the early stages of the transcription process, low levels of RNA promote the formation of transcriptional condensates, whereas during transcription elongation, high levels of RNA contribute to condensate dissolution (25). It is worth noting that the cofactor condensation and triggers post-translational modifications, resulting in the rearrangement of condensate components and release of RNA polymerase (Pol) II from the promoter site into the active elongation stage (26). This process involves the formation of hubs on active genes by RNA Pol II through interactions between the carboxy-terminal domain (CTD) and interactions with activators, and the release of RNA Pol II from the hubs through CTD phosphorylation for promoter escape and transcription elongation (27). Chen et al. (28) identified a novel circular RNA (circRNA), circVAMP3, which interacts with CAPRIN1 under stress conditions to drive LLPS to form stress granules and inhibit proto-oncogene c-MYC translation, thereby downregulating MYC proto-oncogene protein expression. (**Figure 2**)

We have found that numerous studies have focused on LLPS-mediated transcriptional and translational regulation, while research on the regulation of normal protein folding remains underexplored. Due to the central role of transcription in cellular physiological mechanisms and the fact that translational inhibition caused by compartmentalization of specific RNAs in stress granules is the most common feature of membrane-less structures, these factors have attracted greater scientific interest, leading to more concentrated and in-depth research in these areas.

#### 2.1.2 LLPS involves in epigenetic modifications that regulate the activity and accessibility of gene expression

Post-translational modifications of histone tails can modulate changes in the chromatin structure (29). The layered condensate formed by histone modifying enzymes undergo LLPS creates “reaction chambers” around chromatin and enhances catalytic activity in the region of the gene bodies (30). In the novel histone H3-H4 tetramer assembly pathway, the newly synthesized histones H3-H4 undergoes acetylation and ubiquitination. Acetylation and ubiquitination reactions are accelerated by two different enzymatic reaction chambers that promote the deposition of H3-H4 tetramers on the chromatin (31). MeCP2 is thought to recognize DNA methylation and bind to methylated CpG dinucleotides in the DNA (32). *In vitro*, MeCP2 induced nucleosome array compaction and promoted LLPS, whereas DNA methylation further enhanced the formation of MeCP2 chromatin condensates (33). Therefore, modulating epigenetic modifications through LLPS represents a critical breakthrough for altering chromatin conformation and gene accessibility.

#### 2.1.3 LLPS is involved in DDR and contributes to maintaining genetic stability

Although all life forms strive to pass on their genetic material intact, DNA damage - whether originating from endogenous or environmental sources - is inevitable (34, 35). To meet this challenge, cells have evolved sophisticated mechanisms to detect these damages and rapidly signal to initiate the repair process, ensuring the stable inheritance of genetic information (35, 36). This involves the DDR checkpoint pathway, including the ataxia-telangiectasia mutated (ATM), AT and Rad3-related protein (ATR), and DNA-dependent protein kinase (DNA-PK), in which the ATM and ATR pathways can be activated by various stress conditions, such as DNA damage, RNA Pol I inhibition, DNA replication stress, and osmotic stress. Moreover, the ATR pathway can be activated by biomolecular condensation in the absence of perturbations. DNA topoisomerase 2-binding protein 1 (TopBP1) directly activates the ATR-DDR checkpoint pathway. TopBP1 is a direct activator of the ATR DDR checkpoint pathway and triggers ATR via LLPS, whereas molecular condensates formed by APE1 via LLPS activate ATR by recruiting ATR/ATRIP, TopBP1, and ETAA1 (37). Under stress conditions, APE1 regulates the ATR-DDR pathway through its nuclease activity; whereas under unperturbed conditions, overexpressed APE1 activates this pathway in a DNA/RNA-independent manner. This mechanism is directly associated with its NT33 motif, which facilitates the assembly of biomolecular condensates within the nucleolus and mediates the recruitment of ATR-DDR pathway activators, ultimately leading to reduced rRNA transcription and cell cycle arrest, among other outcomes (38).This multi-level activation mechanism ensures that the ATR pathway operates efficiently under different stress conditions, providing cells with comprehensive DNA damage repair capabilities. DNA double-strand break (DSB) is the most serious type of DDR, which can lead to cell death or cancer mutations (39). When DSB occurs, key biomolecules are rapidly and highly recruited to the site of injury, activating downstream signals to initiate the repair process, which is critical for cell survival under severe injury (40).

The role of Fused in sarcoma (FUS) proteins in RNA metabolism is well known, but recent studies have shown that it also has a unique function in the DDR process. It not only enhances the activity and recruitment of DNA repair enzymes, but also interacts with other proteins to regulate DDR signaling pathways. Notably, FUS undergoing LLPS binds to proteins associated with chromatin remodeling and DNA damage and participates in DDR, whereas FUS not undergoing LLPS mainly interacts with factors associated with pre-mRNA processing and regulates gene expression (41, 42). Furthermore, Levone et al. (43) demonstrated for the first time the importance of FUS-dependent LLPS in the early initiation of DDR and in the correct assembly of DSB repair complexes and that FUS is required for the recruitment of the DDR factors KU80, NBS1, 53BP1, and SFPQ (a DDR-associated RNA-binding protein) to the DNA damage site. The fact that the same protein may have multifaceted functions reflects the diversity of protein functions. However, it is the key mechanistic processes that determine these functional differences that are the crucial link, which highlights the importance of LLPS processes in DDR.

### 2.2 Cellular phenotypic regulatory processes involving LLPS

**Figure 3:**
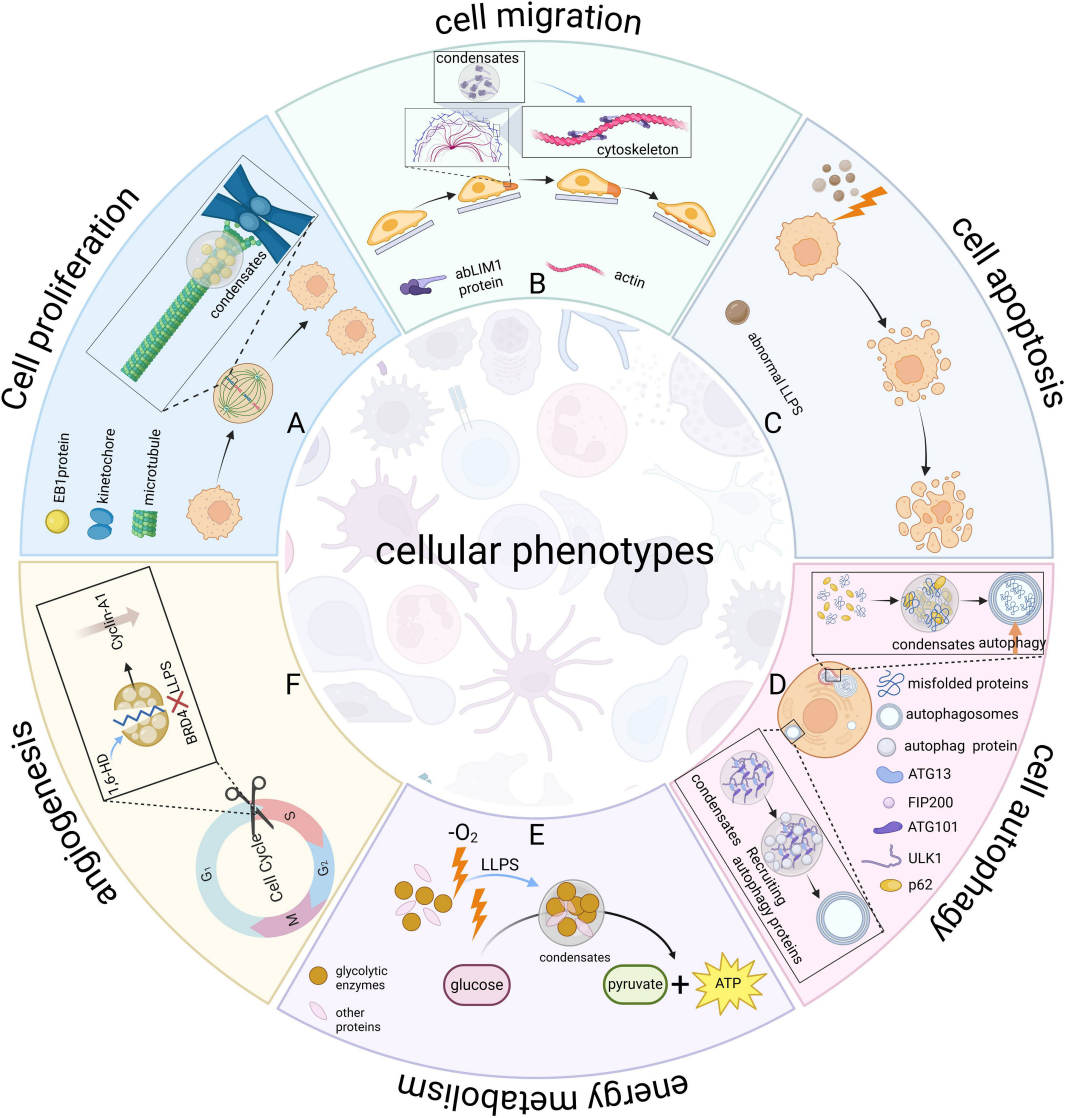
LLPS regulates cellular phenotypes. LLPS serves as a universal organizer of biomolecules, dynamically coordinating spatiotemporal control of essential cellular processes. **(A)** Cell proliferation. EB1 protein forms microtubule plus-end hubs via LLPS, promoting kinetochore-microtubule attachment and precise chromosome segregation, regulating mitosis. This condensation enables the spatiotemporal organization of the spindle assembly core, ensuring precise and accurate cell division. **(B)** Cell migration. The abLIM1 protein forms aggregates via LLPS, assembles actin filaments and networks, builds the cytoskeleton, and regulates cell motility. **(C)** Apoptosis. Aberrant LLPS-mediated signaling pathways can induce apoptosis. **(D)** Autophagy. ATG13 in the ULK1 complex interacts with FIP200 to drive the LLPS of complex and recruits autophagy proteins to form autophagosomes. p62 recognizes misfolded proteins and transports them to specific regions, forming condensates that promote aggregate autophagy. **(E)** Energy metabolism. Glucosomes form condensates under hypoxic stress to colocalize glycolytic enzymes and increase glycolytic rates. **(F)** Angiogenesis. LLPS inhibitor 1,6-HD inhibits BRD4 LLPS, disrupts cyclin A1 expression, affects endothelial G1/S transition, inhibits endothelial cell growth and proliferation, and ultimately impairs angiogenesis. Figure created in BioRender. (2025) https://BioRender.com/l26y961.

Gene expression determines cellular phenotype by controlling the production of functional molecules within the cell (44). LLPS can regulate gene expression by aggregating or segregating specific molecules, thereby affecting cellular phenotypes, including cell proliferation, migration, apoptosis, autophagy, energy metabolism, and angiogenesis (**Figure 3**).

#### 2.2.1 Role of LLPS in cell proliferation

Mitosis is a central process in the proliferation of eukaryotic cells (45–48). LLPS-mediated regulation of chromatin organization and chromosome behavior during mitosis directly affects the efficiency and accuracy of cell proliferation (49). For example, compartments formed by LLPS promote the formation of functional kinetochores and specific biochemical reactions between heterochromatin and kinetochores (50). Besides, LLPS of the chromosomal passenger complex (CPC), which is required for kinetochore localization, regulates chromosome segregation (51). During mitosis, histone labeling promotes the enrichment of CPCs in the inner kinetochore and triggers the formation of condensates via LLPS. Once formed, these condensates further strengthen the interaction between CPCs and histones, thus effectively attracting more CPCs to the region in a stable manner and facilitating the assembly of CPC condensates in the inner kinetochore (52). Another key example, LLPS of end-binding (EB)1, a member of the EB family of proteins, is a key regulator of microtubule plus-end hubs, which couple kinetochore-microtubule attachment to precise chromosome segregation, providing precise and robust spatiotemporal regulation of mitosis (53). Meanwhile, The disordered repeat structural domain of Ki-67 generates a charge blockade by phosphorylation and undergoes LLPS, which is associated with cell cycle specificity (54). Since Ki-67 protein is closely related to cell proliferation and its expression varies with the progression of the cell cycle, it is suggested that Ki-67 may regulate the cell cycle and influence cell proliferation activity through LLPS. In summary, the effects of LLPS on cell proliferation at the three levels of efficiency, accuracy, and activity together reveal the central role of LLPS in the regulation of cell proliferation.

#### 2.2.2 Role of LLPS in cell migration

The actin cytoskeleton is a key structural basis and power source for processes such as cell migration, division and organelle positioning (55, 56). Cells regulate the construction and assembly of the cytoskeleton and its functioning through LLPS, thereby affecting these biological processes. For example, abLIM1 is an actin-binding protein that undergoes LLPS through its dematin-homologous unfolded region, forms aggregates, and self-assembles into bundled actin filaments and networks for cytoskeletal construction (57). In stress responses, the actin nucleation factor DIAPH3 acts as a scaffolding protein and drives LLPS to form DIAPH3 granules, which are central to the regulation of actin cytoskeleton remodeling (58). In addition, the enhanced activity of actin-binding proteins (e.g., N-WASP and Arp2/3) is associated with their integration into signaling protein condensates (56), whereas the phenomenon of LLPS of Nephrin/Nck/N-WASP signaling proteins on membranes prolonged the residence time of the N-WASP and Arp2/3 complexes on membranes and enhanced action assembly (59). LLPS also regulates cell motility by mediating the formation of modular GIT1/β-Pix condensate (60). In summary, LLPS mediates the segregation and cohesion of cytoskeleton-associated proteins, which in turn dynamically reorganizes the cytoskeleton and ultimately affects the efficiency and direction of cell migration.

#### 2.2.3 Role of LLPS in apoptosis and autophagy in apoptosis and autophagy

Both apoptosis and autophagy are programmed cell death processes that ultimately lead to cellular self-eradication (61, 62). Apoptosis is an ordered and genetically regulated cellular suicide process (63). Amino acid starvation-induced LLPS forms the proteasome condensate SIPAN, which triggers P53-mediated apoptosis and reduces cell survival (64). Stress granules formed by LLPS under stress conditions isolate the signaling scaffold protein RACK1, inhibit MAPK apoptotic signaling, and protect cells from apoptosis (65). Besides, Veling et al. (66) found that extreme tolerance-associated proteins reduced apoptosis through LLPS, Based on studies of extremely tolerant organisms and protective proteins. The effect of LLPS on apoptosis presents a seemingly contradictory duality. On the one hand, promotion of apoptosis mediated under specific stressful environments can optimize resource allocation to maintain the overall homeostasis of the organism. On the other hand, inhibition of apoptosis mediated in response to external stresses maintains cell survival, thereby ensuring the functional integrity of tissues and organs. This difference stems from the fact that different physiological and environmental conditions trigger different functional pathways of LLPS, but its core goal is the same - i.e., to protect the organism from internal and external threats.

Autophagy is a process by which cytoplasmic contents such as damaged or redundant organelles and invasive microorganisms are sequestered in double-membrane autophagosomes and subsequently transported to lysosomes for degradation and recycling (67, 68). ATG13 in the mammalian ULK1 complex interacts with FIP200 to drive the LLPS of the ULK1 complex and recruits downstream autophagy proteins to form autophagosomes (69). p62 is a key junction protein in autophagy, and the E3 ligase Smurf1 enhances Nrf2 activation and promotes autophagy by increasing p62 LLPS levels (70). Aggrephagy is a specific type of autophagy that effectively removes protein aggregates and misfolded proteins from cells, and maintains the stability of the internal environment (71). These aberrant deposits cannot be recognized directly by the aggrephagy mechanism, but undergo a series of precise regulations, including recognition, transport, and localization. During this process, the interaction of ubiquitin-binding domains (UBDs) with polyubiquitin ensures correct protein processing. The UBDs of p62 and TAX1BP1 recognize and bind polyubiquitin to misfolded proteins. The labeled proteins are then translocated to specific regions in the cytoplasm, where they form condensates containing high concentrations of ubiquitin via LLPS. These ubiquitin-enriched condensates act as signals for aggrephagy, facilitating the activation and conduct of this degradation pathway (72, 73). In addition, gel-like protein condensates trigger the formation of peripheral autophagosome membranes (74), further illustrating the importance of LLPS in the autophagic process. Taken together, The LLPS-mediated autophagy process consists of two main aspects: 1) assembly of autophagosome-forming sites, and 2) sorting and degradation of protein cargoes. These mechanisms involve the initiation of autophagy and multiple key execution steps, ensuring efficient and precise degradation of intracellular waste materials, thereby maintaining the internal homeostasis and functional integrity of the cell.

#### 2.2.4 Role of LLPS in energy metabolism

LLPS maintains intracellular energy balance and redox homeostasis by regulating glucose and lipid metabolism and responding to redox imbalances. Glucosomes form membrane-free condensates under hypoxic stress to colocalize glycolytic enzymes and increase glycolytic rates (75). The Troyer syndrome-associated protein spartin activates and recruits the HECT-type ubiquitin E3 ligase Itch to form itch condensates via LLPS, a process that promotes the autophagy-dependent turnover of lipid droplets and supports normal lipid metabolism (76). It is worth noting that several energy metabolic pathways, such as glycolysis, the tricarboxylic acid cycle, and oxidative phosphorylation, fall under the redox category. Oxidation-reduction imbalance may lead to oxidative stress and cellular damage, and organisms often maintain redox homeostasis by correcting redox imbalance responses. Post-translational modifications of the low-complexity domains (LCD) of proteins can respond to redox imbalances and change the phase properties of proteins. When the redox imbalance exceeds a certain threshold, cells activate the signal transduction pathways involved in the LLPS response, such as the MAPK cascade, to activate the Nrf2-mediated oxidative stress response (77).

#### 2.2.5 Importance of LLPS in angiogenesis

Jiang et al. (78) first explored the possibility of the involvement of LLPS in angiogenesis using the LLPS inhibitor 1,6-HD. 1,6-HD disrupts cyclin A1 expression by inhibiting phase separation of BRD4, specifically affecting endothelial G1/S phase transition, inhibiting endothelial cell growth and proliferation, and ultimately impairs angiogenesis. Although the role of LLPS in angiogenesis is currently understudied, we still expect more scientists to conduct a more in-depth exploration in this field.

As a key cellular physiological regulatory mechanism, LLPS has a profound impact on cellular physiological processes at multiple levels. These findings provide new perspectives for understanding how cells finely regulate their behavior and offer potential targets for the development of therapeutic strategies against specific cellular physiological abnormalities. Tumors are complex tissues composed of different cell types (79). They can evade growth inhibition and cell death and maintain cell proliferation, leading to cellular immortalization. They also induce angiogenesis, which contributes to tumor growth and spread; meanwhile, infiltrative and metastatic processes further contribute to tumor progression. In addition, metabolic reprogramming and immune escape have been shown to be common features of tumor cells (80). The reason for the differential characteristics of tumor and normal cells is the accumulation of abnormal gene expression (81). Therefore, tumors can also be considered as diseases with gene mutations or dysregulated gene transcription (82). An increasing number of studies have begun to link cancer-related genes to the assembly of abnormally altered condensates and LLPS has emerged as a new perspective for exploring the mechanisms of tumorigenesis, progression and therapy (83). Next, we will summarize these systematically.

## 3 Mechanisms of LLPS in tumorigenesis

### 3.1 Role of LLPS in the process of carcinogenesis by Fos

**Figure 4:**
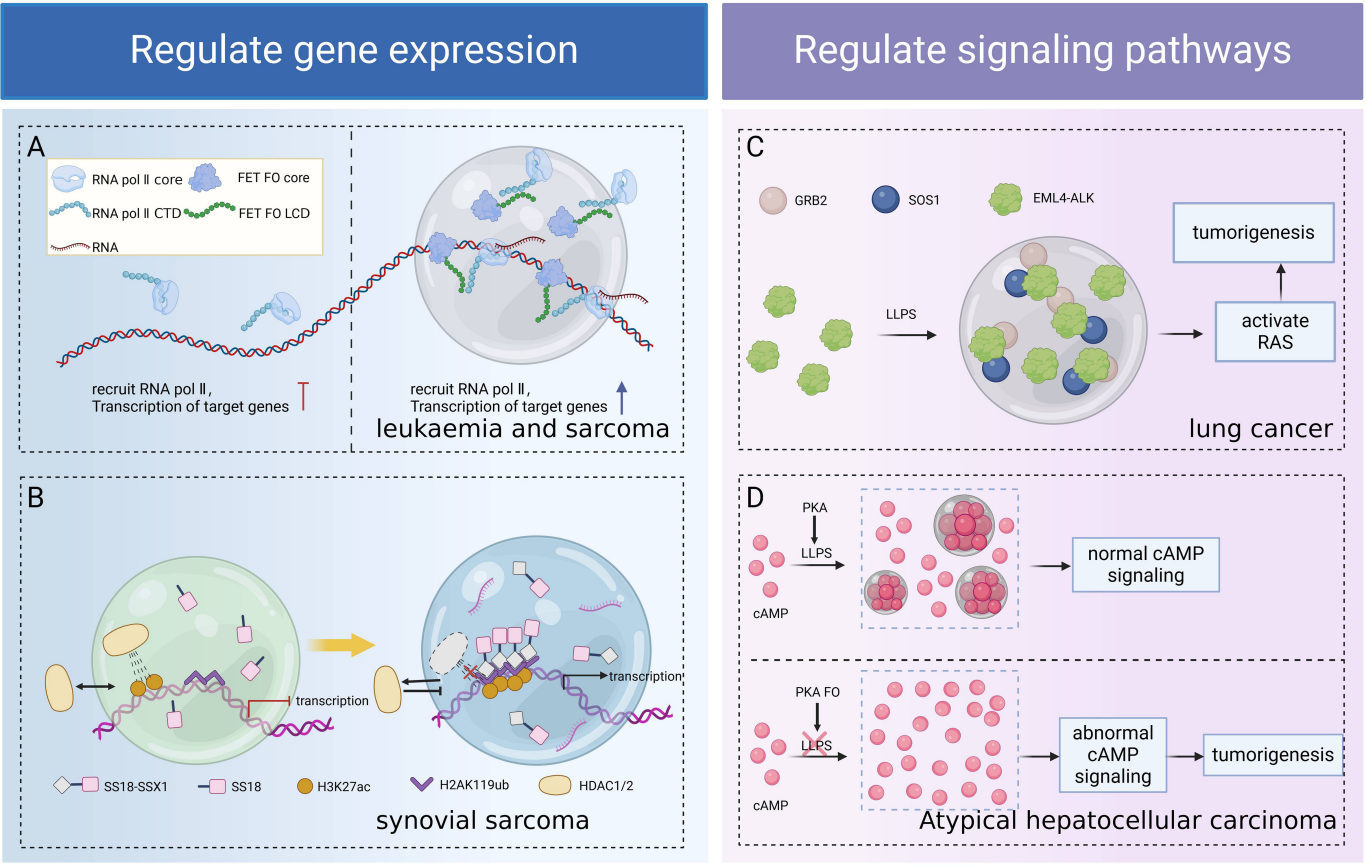
Role of LLPS in tumorigenesis due to FOs. FOs form functional condensates through LLPS, regulating transcription **(A)**, chromatin remodeling **(B)**, and signaling pathways **(C, D)** separately, thereby revealing the universality of LLPS as an oncogenic hub. **(A)** The LCD of FET FO drives LLPS, forms condensates to recruit RNA Pol II, and enhances fusion oncogene transcription, leading to sarcoma and leukemia. **(B)** SS18-SSX1 condensate readily excludes the HDAC1/2 complex, remodeling the genome structure, resulting in the aberrant accumulation of H3K27ac and overexpression of synovial sarcoma target genes. **(C)** EML4-ALK FO forms condensates through LLPS, locally concentrates the RAS activation complex GRB2/SOS1, and activates RAS and MAPK signaling pathways to promote lung carcinogenesis. **(D)** The condensate formed by PKA via LLPS has cAMP and PKA activities that regulate normal cellular activities. PKA FO associated with atypical hepatocellular carcinoma disrupts LLPS, impairs cAMP compartmentalization, aberrant cAMP signaling, and promotes cell proliferation and transformation. Figure created in BioRender. (2025) https://BioRender.com/a81u327.

Both FOs and ectopically expressed normal/truncated oncoproteins have oncogenic potential (84), and their oncogenic mechanisms include activation of aberrant signaling pathways, inhibition of apoptosis, promotion of cell proliferation, modulation of the cell cycle, interference with the DNA repair mechanism, and effect on gene expression (85–88). Among them, FOs encoded by IDR/LCD-containing fusion oncogenes acquired LLPS capability and were able to promote tumorigenesis by regulation of gene expression in the nucleus and signal transduction in the cytoplasm through LLPS (89, 90) (**Figure 4**).

#### 3.1.1 Role of LLPS in the process of FOs induce tumors by regulating gene expression

The LCD of FUS/EWS/TAF15 (FET) FOs favors LLPS, and condensates formed via LLPS recruit RNA Pol II and promote aberrant gene transcription associated with oncogenic transformation in sarcoma and leukemia (91). The SS18-SSX1 condensate readily excludes the HDAC1/2 complex and remodels the genome structure, resulting in the aberrant accumulation of H3K27ac at the chromatin loci, leading to the overexpression of oncogenic target genes and contributing to synovial sarcoma development (92, 93). NUP98-HOXA9 is an IDR-containing TF chimera, and IDR-driven LLPS promotes chromatin occupancy of the chimeric TF, enhancing oncogene expression in a “super enhancers (SEs)” mode and inducing leukemia transformation (18, 90).

#### 3.1.2 Role of LLPS in the process of FOs influencing signaling to induce tumorigenesis

LLPS regulates signaling processes in various ways, including increasing the affinity for protein interactions and tuning biochemical reaction conditions in specific regions (94, 95). The specific compartment formed by LLPS increases the concentration of target proteins, promotes receptor-kinase binding, and hinders competing ligands, thus overcoming the problem of target uncertainty (96). Classical receptor tyrosine kinases (RTKs) signaling occurs at the lipid membrane, whereas new studies have found that certain RTK (including ALK and RET) FOs reassemble into membrane-less protein particles that locally condense the RAS-activated complex GRB2/SOS1, organizing RTK/RAS/MAPK signaling in a lipid membrane-independent manner (97). For example, EML4-ALK FO forms condensates through LLPS, activates the RAS and downstream MAPK signaling pathways, and promotes lung carcinogenesis and malignant transformation (14, 15, 97). The subunit of cAMP-dependent protein kinase PKA, RIα, forms a condensate via LLPS, which has cAMP and PKA activities, to regulate normal cellular activities, whereas PKA FO associated with atypical hepatocellular carcinomas block the LLPS of RIα, which impairs the compartmentalization of cAMP, providing conditions for aberrant cAMP signaling and contributing to cell proliferation and transformation (98).

Although the above studies suggest that FOs involve the LLPS mechanism in enhancing target gene expression and regulating signaling for carcinogenesis, there is a lack of experimental evidence to clearly indicate that LLPS is the sole or key factor in their carcinogenesis, and LLPS may act in concert with other factors to promote the carcinogenesis process. Therefore, future studies need to further explore the interactions and synergistic effects between LLPS and other oncogenic mechanisms.

### 3.2 Role of LLPS in mutant tumor suppressor genes driving carcinogenesis

**Figure 5:**
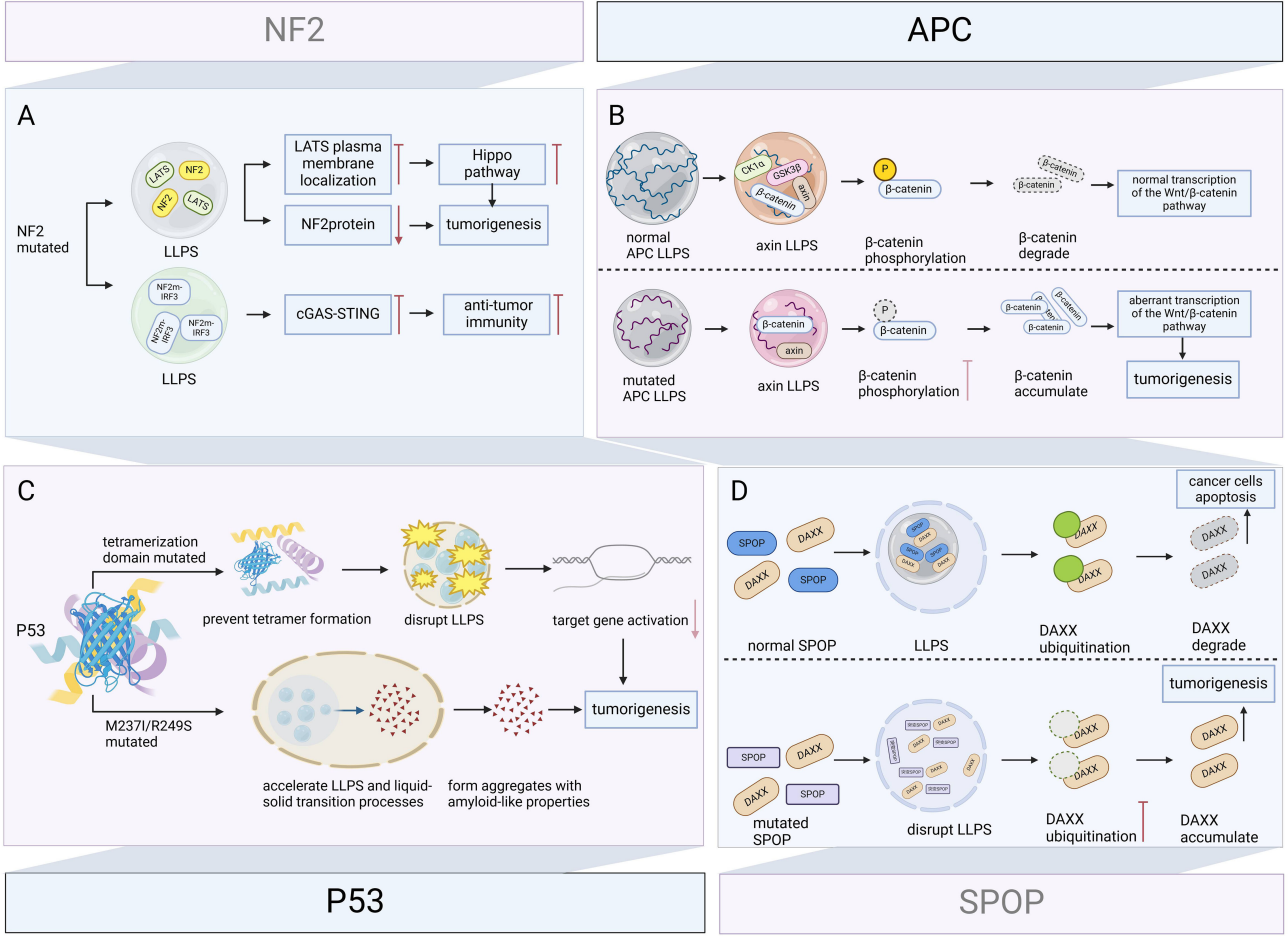
Role of LLPS in mutant tumor suppressor genes driving carcinogenesis. Tumor suppressor genes dynamically regulate key tumor-suppressive or apoptosis-promoting pathways through LLPS. Their mutations disrupt condensate formation and alter phase separation properties, leading to loss of tumor-suppressive function or acquisition of oncogenic activity. **(A)** NF2 mutation inhibits LATS plasma membrane localization via LLPS and inactivate the Hippo pathway, while decreasing NF2 protein levels and promoting meningeal cell proliferation and tumorigenesis. NF2 mutation also inhibits cGAS-STING signaling via LLPS, eliminating anti-tumor immunity, and synergistically promote tumor progression. **(B)** Under physiological conditions, APC promotes axin LLPS, recruits GSK3β, CK1α, and β-catenin, assembles a multiprotein β-catenin destruction complex, and promotes GSK3β-mediated β-catenin phosphorylation and β-catenin degradation. Mutant APCs form condensates of different composition that fail to degrade β-catenin, and the accumulated β-catenin enhances the transcriptional activity of the Wnt/β-catenin signaling pathway, leading to colorectal carcinogenesis. **(C)** Mutation in the tetrameric domain of P53 prevents tetramer formation and disrupts LLPS, reducing target gene activation and carcinogenesis. M237I and R249S mutated P53 accelerates the LLPS and liquid-solid transition process, forms aggregates with amyloid-like properties, and promotes the oncogenic gain-of-function. **(D)** Under physiological conditions, SPOP and DAXX form SPOP/DAXX bodies via LLPS, which reduce DAXX levels by ubiquitinating and induce apoptosis in cancer cells. Disruption of LLPS and DAXX ubiquitination caused by SPOP mutations can drive tumorigenesis. Figure created in BioRender. (2025) https://BioRender.com/l10x566.

Sustaining cell proliferation signaling and evading growth inhibition are important features of cancer (80). Tumor suppressor genes are activated when cells are stimulated by stressors and core cellular stress responses occur, including cell cycle arrest and apoptosis (99). When tumor suppressor genes are mutated or lose their function, they are unable to effectively control cell proliferation, which may lead to unrestricted cell growth and division, and ultimately, the formation of tumors (100). LLPS is involved in oncogenic processes associated with mutant in tumor suppressor genes (e.g., p53, NF2, APC, speckle-type POZ protein (SPOP) etc.) (**Figure 5**).

#### 3.2.1 p53

p53 is an important tumor suppressor, which induces cell cycle arrest, DNA repair, or apoptosis upon binding to target DNA sequences and is central to the maintenance of genomic stability and prevention of tumorigenesis, and its gene TP53 is highly mutated in approximately 50% of human cancers (101, 102). Because p53 itself can form droplets, this suggests that LLPS may regulate the function of p53 (102). Further research confirms this conjecture. Mutation of the tetramerization domain prevents tetramer formation and disrupts disordered unstructured basic region-induced LLPS, reducing target gene activation and causing cancer (103). Furthermore, M237I and R249S also mutated P53 accelerated LLPS and liquid-solid transition processes, tending to form aggregates with amyloid-like properties (104), which are associated with oncogenic gain-of-function (105).

#### 3.2.2 NF2

In neurofibromatosis type 2, the NF2 c.770-784del mutation inactivates the Hippo pathway by inhibiting LATS plasma membrane localization via LLPS. Additionally, this mutation reduces NF2 protein levels, which promotes meningeal cell proliferation and tumorigenesis (106). Furthermore, NF2 alleviates TBK1 inhibition and promotes innate immunity. However, Mutations in the FERM domain inhibit cGAS-STING signaling via LLPS, eliminating antitumor immunity, and related NF2m-IRF3 condensates have been observed in vestibular schwannomas (107).

#### 3.2.3 APC

APC is a tumor suppressor gene with a very high mutation rate in colorectal cancer (108), and its mutation and inactivation are key early events in colorectal cancer tumor formation (109). APC promotes LLPS of axin, which in turn provides a scaffold for the recruitment of GSK3β, CK1α, and β-catenin, driving the composition of the multiprotein β-catenin destruction complex. The β-catenin destruction complex promotes GSK3β-mediated phosphorylation of β-catenin, further degrading β-catenin. Whereas mutant APC is also capable of LLPS, the assembled condensates cannot recruit GSK3β and CK1α, which results in the inability to phosphorylate β-catenin, allowing β-catenin to accumulate in the cytoplasm, further enhancing the transcriptional activity of the Wnt/β-catenin signaling pathway, and ultimately leading to colorectal cancer development (17, 110, 111).

#### 3.2.4 SPOP

SPOP is a substrate junction protein for the cullin-3-RING ubiquitin ligase, and its mutations lead to prostate cancer, gastric cancer, and other solid tumors (112). DAXX is a substrate protein of SPOP that maintains the survival of cancer cells by downregulating the transcription of various tumor suppressors. SPOP is localized in the nucleus of cells with droplet characteristics in membrane-less organelles, and when co-expressed with DAXX, it can form droplet-like SPOP/DAXX bodies via LLPS, which can reduce DAXX levels by ubiquitylating DAXX, effectively inducing apoptosis in cancer cells (11). Our study demonstrates that disruption of LLPS and DAXX ubiquitination caused by SPOP mutations are potential mechanisms driving tumorigenesis (113).

It is worth noting that not all tumor suppressor gene mutations are oncogenic by directly altering the behavior of LLPS; some are due to changes in the proteins involved in LLPS, resulting in the biomolecular condensates being unable to carry out normal physiological functions, thus promoting carcinogenesis. Therefore, the oncogenic mechanisms associated with LLPS should be explored in a multidimensional manner.

### 3.3 Regulation of epigenetic modifications through LLPS drive tumorigenesis

In preceding chapters, we have expounded the significance of LLPS as a pivotal regulatory mechanism in epigenetic modification. It is noteworthy that extensive research has revealed LLPS’s capacity to mediate chromatin remodeling under pathological epigenetic states, thereby establishing itself as a central driver in oncogenic progression.

**Figure 6:**
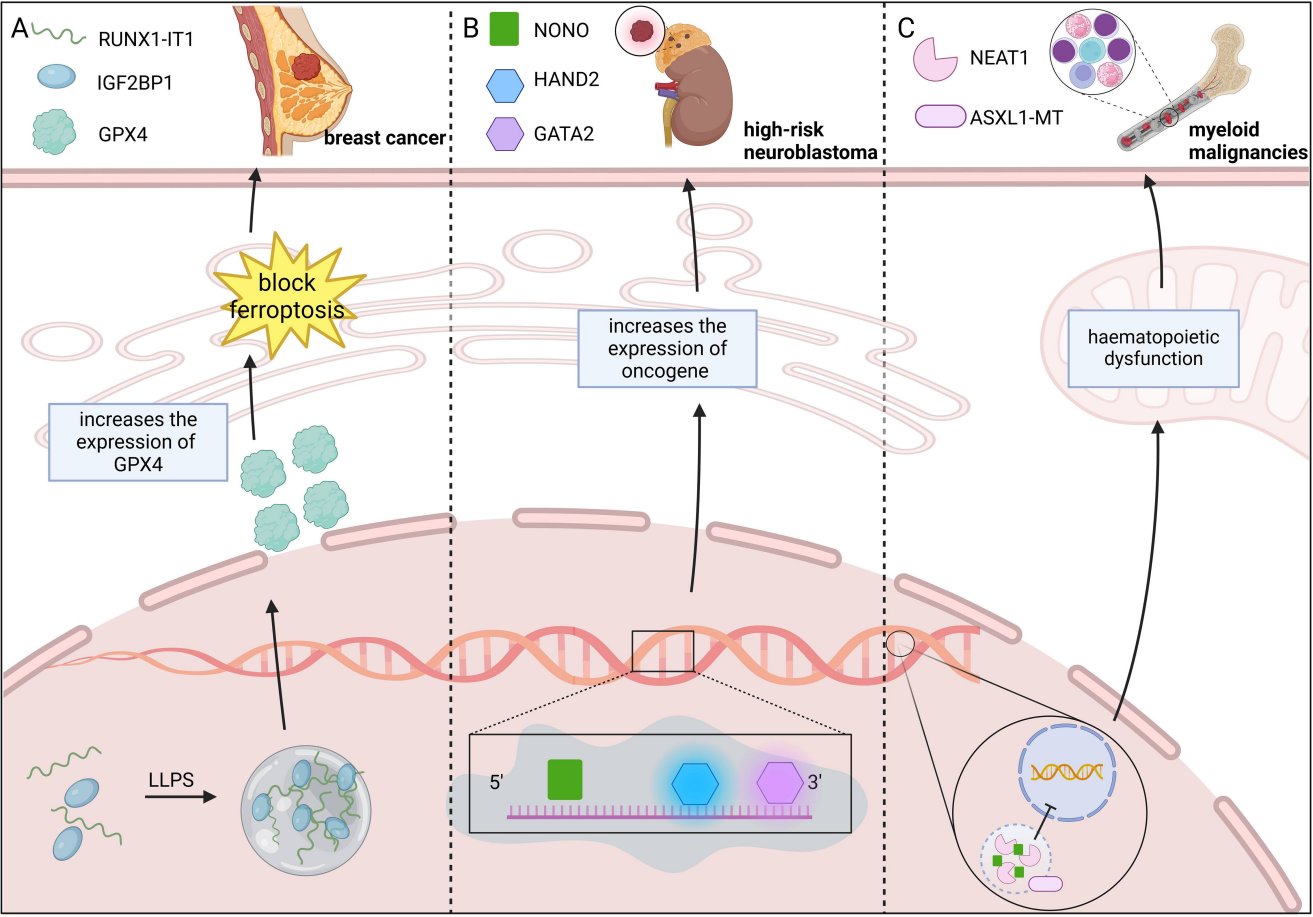
LLPS drives tumorigenesis by regulating epigenetic modifications. **(A)** RUNX1-IT1-IGF2BP1 condensates formed via LLPS suppress ferroptosis and drive breast cancer progression. RUNX1-IT1 forms an IGF2BP1 biomolecular condensate with IGF2BP1, which increases GPX4 expression and blocks ferroptosis, and promotes breast cancer development. **(B)** NONO LLPS enhances oncogene expression, driving high-risk neuroblastoma. NONO binds to RNA and enhances high-risk neuroblastoma oncogene expression by increasing HAND2 and GATA2 processing via LLPS. **(C)** ASXL1-MT mutation disrupts LLPS, leading to myeloid malignancies. ASXL1-MT mutants fail to develop LLPS, disrupting paraspeckle formation and RNA splicing, leading to hematopoietic dysfunction and myeloid malignancy development. Figure created in BioRender. (2025) https://BioRender.com/w40p249.

METTL3 undergoing LLPS can regulate epigenetics by modulating the dynamic assembly of the m6A methyltransferase complex (METTL3/METTL14/WTAP). Han et al. (114) tested relatively common METTL3 mutations in cancer using the Cry2 LLPS system and found that the R415C and E516K mutants were unable to undergo LLPS in the presence of Cry2 fusion and blue-light irradiation, suggesting that the disruption of LLPS resulting from mutations in METTL3 may alter epigenetic inheritance, which may in turn promote tumorigenesis. RUNX1 intronic transcript 1 binds to the N6-methyladenosine m^6^A reader IGF2BP1, promotes the formation of the IGF2BP1 biomolecular condensate, and improves the stability of GPX4 mRNA, which increases the expression of GPX4, blocks ferroptosis, and promotes breast cancer development (16). In multiple myeloma(MM), epigenetic defects are associated with the genomic instability of tumors, anticancer drug resistance, and cancer cell plasticity (115). The RNA-binding protein NONO interacts with the 5’ end of pre-mRNA to initiate pre-mRNA processing. The epigenetic regulator ASXL1 is involved in paraspeckle formation by increasing the level of NEAT1 through LLPS while increasing the binding of NONO to the lncRNA NEAT1. ASXL1 mutations are frequent in myeloid malignancies. The lack of a C-terminal IDR in the ASXL1-MT mutant disrupts paraspeckle formation and RNA splicing, leading to hematopoietic dysfunction (116). In high-risk neuroblastomas, NONO binds to RNA and undergoes LLPS, which increases the processing of the SE-related genes HAND2 and GATA2, ultimately enhancing oncogene expression (117) (**Figure 6**).

### 3.4 LLPS-mediated DDR repair efficiency degradation is a potential mechanism for tumorigenesis

DDR plays a role in protecting genomic integrity and stability, and DDR abnormalities may lead to the inheritance of mutations in proliferating cell populations. There are two main pathways of DDR repair: nonhomologous end-joining (NHEJ) repair and homologous recombination (HR) repair. Ubiquitinated RNF168 undergoes LLPS, which restricts the recruitment of RNF168 to DNA damage sites, resulting in reduced RNF168-catalyzed H2A ubiquitination, further limiting the 53BP1 content of the nuclear condensate, and thus decreasing the repair efficiency of NHEJ (39, 118–120). It is important to note that whether this sequence of events leads to tumorigenesis has not been definitively answered by the current study. Although the reduced efficiency of NHEJ repair resulting from ubiquitinated RNF168 undergoing LLPS does not directly equate to tumorigenesis per se, it does increase the risk of genomic instability, inflammatory response, and imbalance in cell cycle regulation, all of which are closely related to tumorigenesis. Thus, this process may be one of the potential mechanisms of tumorigenesis.

### 3.5 LLPS-mediated tumor immune surveillance and signal transduction

The immune system achieves precise antigen recognition and response within the tumor microenvironment through a multi-layered molecular regulatory network mediated by LLPS. In adaptive immunity, the T cell receptor (TCR) signaling pathway exhibits remarkable antigen discrimination: it demonstrates high sensitivity for recognizing foreign peptide antigens while maintaining weak binding affinity for self-peptides. Mechanistic studies reveal that LLPS, triggered by linker for activation of T cells (LAT) phosphorylation, dynamically recruits key signaling molecules such as PLCγ and Son of Sevenless (SOS) through a nucleation-condensation process. This transmits activation signals to T cells. This LLPS-driven kinetic proofreading system not only ensures selective signal transduction but also endows T cells with the ability to sense cancerous cells in real time (121).Similarly, the formation of B-cell immune synapses relies on LLPS regulation. B cells modulate transitions between different synaptic modes by adjusting the degree of phase separation through normal pulling forces, thereby optimizing spatial resolution during antigen recognition (122). In innate immunity, the cyclic GMP-AMP synthase-stimulator of interferon genes(cGAS-STING)pathway, a core DNA sensing mechanism, undergoes multi-level regulation by USP15 deubiquitination: USP15 activates cGAS via deubiquitination and feeds back positively to promote its dimerization and phase separation. This significantly enhances DNA damage response sensitivity, enabling immune cells to rapidly amplify innate immune signals even in the absence of sustained stimuli (123).

In summary, a thorough elucidation of the pivotal role that LLPS plays across various carcinogenic processes is crucial for unraveling the nature of cancer.

## 4 Role of LLPS in tumor progression

### 4.1 LLPS-mediated regulation of tumor proliferation and metastasis

Histone methyltransferase EZH2 is overexpressed in lung cancer cells. N-terminal glycine myristoylation confers hydrophobicity to EZH2, which drives LLPS of EZH2 in lung cancer cells, compartmentalizes the substrate STAT3, and activates STAT3 signaling, ultimately facilitating rapid lung cancer cell proliferation (124). Abnormal overexpression of methyltransferase-like protein 3 (METTL3) plays an important role in the progression of acute myeloid leukemia (AML). TF YY1 interacts with HDAC1/3 and promoted METTL3 expression and AML cell proliferation via moderate LLPS (125). Alternative polyadenylation (APA) is a regulatory mechanism of gene expression that promotes cancer cell proliferation and migration by shortening the 3’untranslated regions (UTRs) of mRNA. Interestingly, the key factor CPSF6 is usually highly expressed in tumors and is involved in 3’mRNA cleavage and polyadenylation processing, leading to lengthening of 3’UTRs. Further studies revealed that the LLPS of CPSF6, but not its expression level, was the main factor regulating APA in cancer cells. Reducing CPSF6 LLPS leads to shortening of 3’UTRs of cell cycle-related genes and accelerates cell proliferation (126). Histone lysine methyltransferase 2D (KMT2D) is associated with tumorigenesis and metastasis, and is present in a wide range of cancers. The LLPS microenvironment driven by the two LCDs of KMT2D facilitates H3K4 histone monomethylation transcription as well as the expression of the tumor-associated TFs LIFR and KLF4, promoting tumor progression (127). Under hyperosmotic stress, the YAP condensate compartmentalizes TEAD1 and other coactivators to induce YAP-specific proliferative gene transcription (128). YAP is a powerful driver of hepatoblastoma (HB), and coiled-coil domain-mediated LLPS can form active transcription sites that recruit TEAD4, promote oncogene transcription and HB proliferation, and induce chemoresistance in HB (129). In summary, LLPS-mediated regulation of key protein and gene expression promotes tumor cell proliferation and migration.

### 4.2 LLPS-mediated autophagy dysregulation drives tumor progression

Dysfunctional autophagy has been linked to cancer, neurodegenerative diseases, and diabetes mellitus (130). In colorectal cancer, high expression of Mex-3 RNA-binding family member A (MEX3A) is associated with poor prognosis. Processing bodies (PBs) are membrane-free organelles formed by RNA-binding proteins and RNA. MEX3A orchestrates the recruitment of PB components, and the MEX3A/circMPP6 complex dynamically regulates PB function and promotes UPF-mediated phosphodiesterase 5A mRNA degradation. This regulatory mechanism promotes colorectal cancer progression by inhibiting autophagy (131).

### 4.3 LLPS is an important way for tumors to achieve metabolic reprogramming

Cancer cells often undergo metabolic reprogramming to meet the metabolic demands of growth, proliferation, and migration (132). Sestrin2 (SESN2) and insulin-like growth factor 2 mRNA binding protein 3 (IGF2BP3) compete for binding to the 3’UTR region of HK2 mRNA. The formation of stress granules between IGF2BP3 and HK2 mRNA via LLPS stabilizes HK2 mRNA, whereas SESN2 destabilizes HK2 mRNA through an Nrf2/atf4-dependent mechanism. During glucose shortage, hepatocellular carcinoma cells regulate HK2 levels by elevating SESN2 expression, enhancing SESN2 cytoplasmic localization, and decreasing HK2 mRNA half-life, ultimately suppressing glycolysis (133). In addition, cancer cells can increase enzymatic efficiency through compartmentalization of metabolic enzymes to produce more energy to meet metabolic demands, a process that is closely related to LLPS (134). DAZ associated protein 1 (DAZAP1) is significantly upregulated in oral squamous cell carcinoma (OSCC), and clinical samples have shown that high expression of DAZAP1 and cytochrome-c oxidase 16 (COX16) is associated with poor patient prognosis. DAZAP1 accumulates in the nucleus through LLPS, regulates the selective splicing of pre-mRNA, enhances COX16 expression, and enhances mitochondrial metabolism, which ultimately promotes the invasion and metastasis of OSCC (135). LLPS facilitates the metabolic rate of cancer cells, a process that inevitably generates large amounts of free radicals such as reactive oxygen species and reactive nitrogen species. It is well known that LLPS is a key way for normal cells to maintain redox balance. However, for cancer cells, it is a question that deserves to be explored in depth whether they will adopt specific compensatory mechanisms to cope with this challenge in the face of the large amount of free radicals brought about by this highly efficient metabolism.

### 4.4 LLPS-mediated tumor immunosuppression and immune evasion

LLPS exhibits dual regulatory properties in tumor immunity. On one hand, LLPS-mediated dynamic assemblies play a critical role in regulating innate immune responses. Conversely, tumor cells hijack this mechanism to construct immunosuppressive microenvironments. For example, NF2 mutations promote immune evasion by driving abnormal co-localization of the mutated FERM domain with IRF3/TBK1, forming pathological condensates containing PP2A complexes. This aberrant phase separation inhibits TBK1 activation, significantly impairs cGAS-STING signaling, and ultimately blocks STING-mediated antitumor immune responses within tumor cells (107).During PCa progression, upregulated YY1 protein contributes to M2 macrophage polarization. YY1 complexes mediate LLPS, promoting IL-6 expression in M2 macrophages. This phase separation-mediated epigenetic regulatory network suppresses T lymphocyte activity, thereby establishing an immunosuppressive microenvironment (136). Similarly, YAP proteins form transcriptional co-activator complexes via LLPS, facilitating tumor cell immune evasion and inducing resistance to anti-PD-1 therapy (137).

### 4.5 Alternative ways in which LLPS regulates tumor progression

In addition to the above mechanisms that promote tumor progression, LLPS is also involved in regulating gene expression, altering the tumor microenvironment, and promoting angiogenesis, among other ways to drive tumor progression. MTAR1 recruits IGF2BPs to the PABP1-mediated LLPS complex and enhances the stability and translation of MYC mRNAs, thereby promoting tumor progression (138). In addition, MYC may bind to SEs to form LLPS-associated transcriptional condensates, thereby promoting vascular endothelial growth factor (VEGF) expression and angiogenesis (139). Large SEs have been identified around important oncogenes, providing a platform for the LLPS of key TFs and for enhancing target gene expression (140). OCT4, a TF that can form complexes with other TFs (e.g., forkhead box protein A1 (FOXA1), androgen receptor (AR), and nuclear respiratory factor 1 (NRF1)), promotes prostate cancer (PCa) progression through epigenetic alterations and acquires chemotherapy resistance (140, 141). Tumor-associated M2 macrophages and their YY1 are highly expressed, and LLPS of the YY1 complex upregulates interleukin (IL)-6 levels in the tumor microenvironment by promoting IL-6 enhancer-promoter interactions, leading to PCa progression (136). These studies further emphasize the multidimensional role that LLPS plays in tumor progression.

LLPS is involved in both regulating cellular physiology and is closely associated with tumorigenesis and progression. Considering the two-sided role of LLPS in maintaining cellular homeostasis, how to inhibit tumor progression while avoiding adverse effects on normal cells is also an important challenge for future research.

## 5 LLPS in tumor treatment

### 5.1 Intervention in abnormal LLPS may be a potential tumor treatment strategy

LLPS-mediated STAT3 phosphorylation is a key component of EML4-ALK fusion oncoprotein oncogenesis, whereas the pro-carcinogenic effect of EML4-ALK21S variant FOs incapable of LLPS was significantly reduced, suggesting that LLPS, which mediates STAT3 phosphorylation, can be disrupted to treat EML4-ALK-positive lung cancer (15). Aminocyclopropenone 1n (ACP-1n) disrupts the LLPS ability of BRD4 and attenuates BRD4-mediated MYC gene expression, thereby reducing the nuclear size of colorectal cancer cells, decreasing the expression of the nuclear pore protein NUP210, and ultimately slowing down the growth of colorectal cancer (142). MicroRNAs (miRNAs) are post-transcriptional regulators of gene expression involved in several cancer processes in cancer (143). MiRNA-mediated gene silencing recognizes target-repressed mRNAs via miRNA and facilitates accelerated deadenylation in an LLPS-dependent manner (144). Qin et al. (145) first found that miRNA-induced silencing complex (miRISC) exhibits liquid-like properties in colon cancer cells and under LLPS conditions when overexpressed. They further revealed that miR-490-3p significantly reduced the expression of the cell cycle regulatory protein CDK1 at the RNA and protein levels and inhibited the proliferation of colon cancer cells. The LLPS inhibitor 1,6-HD abrogated these effects. Furthermore, this study was performed without overexpressing any other miRISC components, which strongly confirms the mechanism by which miRNA silences gene expression in an LLPS-dependent manner. AR and its splice variants, AR-SVs, initiate transcriptional reprogramming via LLPS and promote PCa progression. UT-143, a small-molecule selective AR-irreversible covalent antagonist, binds to C406 and C327 in the AF-1 region and inhibits AR target gene expression, PCa cell proliferation, and tumor growth (146). LLPS is an important mechanism of tumorigenesis and progression, and small molecule agents are able to specifically target interventions in these processes and are considered as one of the most suitable forms of drugs.

The study reveals that a significant number of FOs form condensates in the nucleus or/and cytoplasm of the cell, which can cause cancer (90). At the same time, antitumor drugs have been observed to aggregate in specific protein condensates *in vitro* and in tumor cells, a process based solely on the physicochemical properties of the drugs rather than their targets (147). This suggests that both the generation of oncogenic drivers and efficacy of antitumor drugs are closely related to biomolecular condensate mediators. This mechanistic understanding provides a promising therapeutic strategy: targeting either condensate dynamics or upstream regulatory nodes enables precise intervention in pathological phase separation, demonstrating broad potential for innate immune activation, adaptive immune regulation, and combination therapies. Designing small molecules or peptides to modulate the dynamic assembly of condensates has thus emerged as a critical approach for such targeted intervention. For example, Thiyagarajan and colleagues (146) successfully developed specific antagonists by elucidating the LLPS properties of the intrinsically disordered transactivation domains of the AR and AR-SV, opening a new avenue for treating advanced PCa. Within adaptive immunity, LLPS influences T cell function through multiple mechanisms. The LLPS-driven dynamic proofreading system confers high selectivity to the TCR signaling pathway, enabling sensitive recognition of foreign antigens while maintaining weak binding to self-antigens (121). Functioning as a molecular “switch” for TCR signalosome assembly, LLPS directly impacts T cell activation and effector functions by regulating condensate formation and dissolution (148). Furthermore, LLPS facilitates energy concentration for initiating TCR signaling cascades, with mechanical forces shaping signal transduction efficacy. Insights into condensate dynamics have consequently provided a theoretical foundation for innovations in T cell vaccines, CAR-T therapies, and adoptive immunotherapies (149). Compared to directly targeting phase separation dynamics, regulating upstream nodes of LLPS can produce cascading amplification effects. For instance, Zhou and colleagues (150) developed the oligonucleotide Svg3 to enhance cGAS LLPS, significantly boosting IFN-I responses. This strategy demonstrated superior antitumor efficacy and pharmacokinetic stability relative to traditional STING agonists, offering a novel approach for combinatorial immunotherapy. Similarly, inhibition of the demethylase KDM4A promotes the formation of liquid-like HP1γ condensates, inducing DNA replication stress and activating the cGAS-STING pathway. The KDM4A inhibitor KDM4i exhibits potent monotherapy effects and overcomes resistance to PD-1 blockade when combined with anti-PD-1 therapy, synergistically suppressing the progression and metastasis of squamous cell carcinoma (SCC) (151).

Collectively, these studies reveal the multidimensional value of LLPS regulation in cancer immunotherapy: spanning direct condensate intervention, upstream targeting, monotherapies, and combination approaches. Future research should prioritize elucidating the spatiotemporal specificity of distinct phase separation events and exploring biophysically informed, personalized therapeutic combinations to fully unlock the clinical potential of LLPS modulation.

In addition, intervening in processes such as autophagy and metastasis associated with tumor progression can prevent progression. RB1CC1/FIP200 is an important autophagy protein, and K276 is the major CREBBP acetylation site of RB1CC1, as well as the ubiquitination site; the acetylation of this site reduces ubiquitination and inhibits the ubiquitin-dependent degradation of RB1CC1. RB1CC1’s CREBBP acetylation, as well as IDR region-mediated LLPS, maintain typical autophagy in breast cancer cells. Furthermore, CREBBP inhibitors reduce RB1CC1 spot formation in addition to inducing RB1CC1 degradation, suggesting that RB1CC1 inhibitors reduce autophagy in breast cancer cells and that there is some effect on LLPS; however, whether this is through an effect on LLPS to reduce autophagy requires further experimental demonstration (152). Certain tyrosine kinase inhibitors (TKIs), such as gefitinib, can initiate a lysosomal stress response to restrict cell motility, which strongly inhibits cell metastasis and invasion in lung metastasis models. Gefitinib induces the formation of p62/NBR1 droplets from SQSTM1/p62 and NBR1, and cellular inhibitors of apoptosis protein 1 in p62/NBR1 droplets accelerate the degradation of Ras-related C3 botulinum toxin substrate 1, ultimately limiting cell movement (153).

### 5.2 LLPS-mediated radiotherapy resistance in cancer

Aberrant activation of the DDR signaling pathway in tumor cells impairs the therapeutic effect of DNA damage and leads to radioresistance (154). NONO is an important tumor radiotherapy resistance regulator that recruits EGFR and DNA-PK and promotes their interaction through LLPS, activates DNA damage-induced T2609-DNA-PK phosphorylation, enhances NHEJ-mediated DNA damage repair, and promotes drug resistance (155). *In vitro* experiments have shown that the nucleolar protein NOP53 enhances the resistance of colorectal cancer to radiation therapy via LLPS. When NOP53 was silenced, not only was tumor growth inhibited, but sensitivity to radiotherapy was also increased. Clinical data further support this finding that patients with high NOP53 expression showed greater resistance to radiotherapy when receiving (156). Although studies directly on NOP53 and radiotherapy resistance have not explicitly addressed the DDR signaling pathway, it is reasonable to speculate that the DDR signaling pathway may play an important role in this process based on the results of the available studies.

### 5.3 LLPS-mediated chemotherapy resistance in cancer

Tumors are resistant to chemotherapeutic agents for various reasons, including alterations in drug transport and metabolism, enhancement of DNA repair mechanisms, epigenetic alterations, and microenvironmental influences (157–159). LLPS promotes tumor drug resistance by altering epigenetics. Nuclear receptor-binding SET domain protein 2 (NSD2) interacts with steroid receptor coactivator-3 (SRC-3) to mediate SRC-3 LLPS, remodeling MM cell chromatin, enhance histone H3 lysine 36 dimethylation of anti-apoptotic gene promoters, and promote MM chemoresistance to bortezomib (BTZ). Interference with NSD2-mediated SRC-3 LLPS may be a therapeutic target for MM. The SRC-3 inhibitor SI-2 disrupts the interaction of SRC-3 with NSD2 and promotes SRC-3 degradation, which enhances the therapeutic sensitivity of MM to BTZ (19). OCT4 is a key transcription factor in the pathology of advanced PCa that is recruited to specific genomic loci and activates other TFs. On the one hand, OCT4 enhances the formation of complexes activating the AR/FOXA1 axis and facilitating the progression of castration-resistant prostate cancer (CRPC); on the other hand, OCT4 forms a complex with NRF1 allowing neuroendocrine prostate cancer (NEPC) to acquire chemotherapy resistance in the absence of AR. The use of ribavirin to disrupt TF collaboration and inhibit the growth of drug-resistant PCa tumors is a typical example of LLPS-based therapy for drug-resistant tumors (141).

There is ample evidence that LLPS is a potential therapeutic target for tumors. Given the close relationship between LLPS and cellular life activities, we hypothesized that if the same LLPS factor changes are observed in different tumor types, then there may be a common mechanistic link between these tumors. For instance, in colorectal cancer, high expression of RNF168 Sentrin/SUMO-specific protease 1 (SENP1) reduces RNF168 LLPS levels and enhances the resistance to DNA-damaging agents. Therefore, SENP1 may be a potential therapeutic target for chemoresistance in colorectal cancer (118). Because of RNF168 and SENP1 are highly expressed in many cancer types, and SENP1 is associated with drug resistance in several cancers, including PCa, leukemia, ovarian cancer, and non-small cell lung cancer (160, 161). Whether SENP1 may be a key target for overcoming chemoresistance in multiple tumors is a question that deserves to be explored in depth in the future, which opens up new possibilities for the development of broad-spectrum anticancer therapies.

### 5.4 LLPS-mediated reversal of drug resistance in cancer immunotherapy

Immunotherapy has emerged as a pivotal modality in oncology, with its core research focus—immune checkpoint blockade—representing a cutting-edge frontier in cancer immunotherapy (162). However, challenges associated with primary, adaptive, and acquired resistance have become critical bottlenecks limiting the clinical efficacy of this approach (163). Notably, recent studies have revealed that aberrant accumulation of biomolecular condensates within the immunosuppressive tumor microenvironment plays a pivotal role in therapy resistance. Such LLPS phenomena regulate tumor and immune cell functions, shape the immunosuppressive niche, and significantly contribute to immunotherapy resistance. For instance, IFN-γ promotes YAP LLPS-mediated condensate formation, assembling transcriptional regulatory hubs that enhance target gene expression and drive PD-1 therapy resistance in tumor cells (137). On exhausted T cells, the inhibitory receptor LAG3 interacts with the TCR-CD3 complex, inducing dissociation of co-receptor-Lck and suppressing TCR signaling through an MHC II-independent mechanism. Remarkably, this receptor-co-receptor interaction involves LLPS and formation of multimeric protein assemblies (164). Given the central role of LLPS in mediating immunotherapy resistance, targeting these condensates represents a promising strategy. KAT8-IRF1 LLPS, for example, enhances PD-L1 transcription by acetylating IRF1 at K78, thereby boosting its promoter binding. Disrupting this condensate with peptide 2142-R8 not only reduces PD-L1 expression but also synergizes with anti-PD-1 therapy *in vitro* and *in vivo (* 165). Xing et al. (166) further demonstrated that cationic polymers induce RNA LLPS, enriching TGF β1 mRNA in condensates to suppress its translation, alleviate immunosuppression, and exhibit potent antitumor effects in breast cancer models. Collectively, strategies disrupting resistance-promoting LLPS or leveraging LLPS to downregulate therapeutic targets hold transformative potential for overcoming immunotherapy resistance.

### 5.5 Development of a tumor prognosis prediction model based on LLPS

Researchers have developed predictive models through database analysis, with the majority validated via cell experiments and clinical specimen testing to confirm their accuracy and efficacy. These models not only facilitate patient prognosis prediction but also demonstrate treatment response evaluation capabilities in select cases, particularly showing practical potential in predicting immune therapy outcomes. This provides critical decision-making references for individualized clinical treatment planning (**Table 1**). For example, Liu et al. (167) retrieved LLPS-related genes from the DrLLPS database and constructed a prognostic signature of LLPS-related genes in skin cutaneous melanoma (SKCM) based on a comprehensive analysis of The Cancer Genome Atlas (TCGA) and Gene Expression Omnibus (GEO) databases, verifying the role of the key gene TROAP in melanoma by *in vivo* and *in vitro* experiments, which independently predicted the survival of melanoma patients. You et al. (168) established a new LLPS-related index based on six DELRGs (FUS, CBX2, TPX2, TAZ, USH1C, and AXIN1) to predict the rate of biochemical-free recurrence of PCa and experimentally verified that FUS regulates the proliferation, migration, invasion, and apoptosis of PCa cells. In addition, the LLPS-associated gene model allows personalized prognostic assessment of patients with epithelial ovarian cancer (169), low-grade glioma (LGG) (139), gliomas (170), lung cancer (171), hepatocellular carcinoma (HCC) (172, 173), clear-cell renal cell carcinoma (ccRCC) (174), bladder cancer (175), digestive system neoplasms (176), breast cancer (177), and endometrial cancer(EC) (178).

**Table 1:** Studies of LLPS-based tumor prognosis predictive models.

Diseases	Data sources	Biomarker/Evaluation indicators	Study stage	Application of models	References
PCa	TCGA,GEO,PhaSepDB, CellMiner ,UALCAN	FUS,CBX2,TPX2,TAZ,USH1C, AXIN1	Basic research:RWPE-1 , 22RV1 , DU145,PC-3 cell lines;benign prostatic hyperplasia,PCa tissue samples	Predicting the prognosis; Predicting the anti-cancer drug sensitivity	(168)
SKCM	DrLLPS,TCGA,GTEX,GEO,cBioPortal	MLKL ,PARVA , PKP1 , PSME1 , RNF114, TROAP	Basic research: A375 , WM-115 ,HaCaT cell lines;Melanoma tissues and adjacent samples	Predicting the prognosis; Predicting the immunotherapy response	(167)
bladder cancer	TCGA,GEO,ArrayExpress,DrLLPS	LLPS-related risk score	Basic research: bioinformatic analysis	Predicting the immunotherapy response	(175)
digestive system neoplasms	TCGA	BRD4 , FBN1 , TP53	Basic research: bioinformatic analysis	Predicting the prognosis; Predicting the drug response; Predicting the immunotherapy response	(176)
LGG	TCGA,the Chinese Glioma Genome Atlas (CGGA),Rembrandt,DrLLPS	LLPS-related prognostic risk score	Basic research: HA1800,U87 , A172 , LN229 , U251, U373 cell lines; LGG clinical samples	Predicting the prognosis; Predicting the immunotherapy response	(139)
HCC	TCGA,GEO,National Omics Data Encyclopedia repository	ZNF32-AS2	Basic research: HEK-293 T,HCC cell lines ;tumor and neighboring non-tumor samples	Predicting the prognosis; Predicting the sensitivities of patients to drugs; Predicting the immunotherapy response	(173)
lung squamous cell carcinoma	TCGA, PhaSepDB,GEO	risk index	Basic research: bioinformatic analysis	Predicting the prognosis	(171)
HCC	TCGA, PhaSepDB ,International Cancer Genome Consortium	LLPS-related gene-based risk index	Basic research: bioinformatic analysis	Predicting the prognosis	(172)
gliomas	Chinese Glioma Genome Atlas,DrLLPS	FABP5	Basic research: LN229 ,U87 cell lines;glioblastoma tissue,pathological slides	Predicting the prognosis	(170)
ccRCC	TCGA,DrLLPS,ArrayExpress	CLIC5 , MXD3 , NUF2 , PABPC1L , PLK1	Basic research: OSRC-2,Caki-1,786-O cell lines; human renal cancer tissues ,adjacent normal renal tissues	Predicting the prognosis	(174)
breast cancer	PhaSepDB,TCGA,cBioportal ,GEO	prediction model	Basic research: bioinformatic analysis	Predicting the prognosis	(177)
EC	GEO,DrLLPS,UCSC	EIF2S2,SNRPC,PRELID1 ,NDUFB9	Basic research: EC tissue ,healthy uterine tissue	Predicting the prognosis	(178)

### 5.6 Advances in LLPS-dependent antitumor drugs

LLPS-dependent antitumor drugs have been studied in many common cancers, such as lung, colon, breast, and prostate cancers. Antitumor effects are exerted in various ways such as regulating oncogene expression, enhancing chemotherapeutic drug sensitivity, improving the microenvironment, and activating antitumor immunity by disrupting the LLPS process or the formation of biomolecular condensates. Nevertheless, most studies are still in the experimental research stage and are dominated by animal and cellular experiments (19, 141, 142, 146, 166, 179–181).

Although tamoxifen has been used in the clinical treatment of estrogen receptor-positive breast cancer, it was initially used on the therapeutic principle of *competitively binding to the estrogen receptor - blocking estrogen action* - *inhibiting tumor cell growth*. Researchers studying the relationship between the condensates and small molecule cancer therapeutics have found that it may also work through the pathway of *reducing ERα enrichment in MED1 condensate* - *downregulate the expression of the MYC oncogene* (147). This finding reveals the possibility of LLPS as a potential therapeutic target, and may hide new pathways to enhance efficacy through modulation of LLPS, even in drugs that already have a clear therapeutic mechanism (**Table 2**).

**Table 2:** Studies of LLPS-dependent antitumor drugs.

Drugs	Cancers	Experimental methods/application phases	Therapeutic targets	Mechanisms	References
SARICA	PCa	animal experiment: Sprague-Dawley rats; cell experiments: LNCaP,COS7,22RV1,LNCaP-AR cell lines	AR AR-SVs	Disrupts LLPS and condensate formation and inhibits AR target gene expression	(146)
Ribavirin	CRPC; NEPC	animal experiment: BALB/c nude mice; cell experiments: DU145,PC3,22Rv1,LNCaP,DU145-CR,PC3-CR cell lines;human primary CRPC/NEPC tissues	OCT4 AR FOXA1 NRF1	Disruption of OCT4-TF complex formation, inhibits CRPC oncogenic transcription and NEPC chemoresistance	(141)
SI-2	MM	animal experiment: NOD.*Cg-Prkdc^scid^Il2rg^tm1Wjl^*/SzJ mice; cell experiments: MM.1R,U266,RPMI-8226,MM.1S,NCI-H929,ANBL-6,ARP-1,CAG,OPM-2,HEK293T cell lines	NSD2 SRC-3	Disruption of NSD2-SRC-3 interaction, inhibits SRC-3 LLPS and enhances BTZ sensitivity	(19)
GSK-J4	osteosarcoma	animal experiment: BALB/c nude mice; cell experiments: Human osteosarcoma cell lines (143B,SJSA1,U2,MNNG,ZOS,ZOSM)	core regulatory circuitry (CRC)	Disrupts CRC LLPS, inhibits oncogenic transcription, cancer cell metastasis, and enhances chemosensitivity	(181)
Elvitegravir	lung cancer	cell experiments: H1299,H1838,A549,H1975,H1650,MCF7,Lncap,H2052,SF268 ,Beas-2B,293FT cell lines	SRC-1 YAP TEAD	Disruption of SRC-1 LLPS in SRC-1/YAP/TEAD condensate and inhibition of YAP carcinogenic transcription	(180)
1,6-HD	pancreatic cancer	animal experiment: nude mice; cell experiments: HPDE6-C7,BxPC-3,PANC-1,AsPC-1,CFPAC-1 cell lines	MYC	Impacts the tumor microenvironment, downregulates MYC oncogene expression, and induces cancer cell death	(179)
ACP-1n	colon cancer	cell experiments: HEK293T,SAS,SW480,SW620 ,HCT116,ccd18co cell lines	BRD4 MYC	Targeting BRD4 LLPS and inhibiting BRD4 assembly-driven MYC expression	(142)
Cationic polymers (PEI,cDex ,DETA-Dex)	breast cancer	animal experiment: BALB/c (nude) mice; cell experiments: 4T1 cell lines	TGFβ1	Induction of RNA LLPS, down-regulation of TGFβ1 expression, activation of anti-tumor immunity, and inhibition of tumor growth	(166)
Tamoxifen	estrogen receptor-positive breast cancer	clinical application; cell experiments: TamR7 (ECACC 16022509).V6.5 murine embryonic stem cells,MCF7,HCT116 cell lines	MED1 ERα	Reduced ERα enrichment in MED1 condensate and reduced MYC oncogene expression	(147)

In summary, LLPS demonstrates multidimensional application potential in oncology. Firstly, targeted therapeutic strategies based on LLPS provide crucial theoretical foundations for developing novel antitumor therapies. Secondly, mechanism-based studies of LLPS in tumor drug resistance have identified new avenues for reversing treatment resistance, offering innovative solutions to overcome existing therapeutic limitations. Furthermore, LLPS-derived prognostic prediction models significantly enhance the precision of individualized treatment planning. Future investigations should prioritize elucidating LLPS regulatory networks and accelerating the development of specific small-molecule inhibitors, thereby propelling cancer therapy toward more precise and efficacious paradigms.

## 6 Prospects

Classical mechanisms of tumorigenesis are mostly attributed to genetic mutations. However, more and more studies have found that LLPS abnormalities in IDRs of proteins can also trigger tumors and promote their progression. These studies provide us with clues to explore the mechanisms of tumorigenesis and progression from a new perspective, rather than being confined to a single focus on gene mutations in the classical theory, which ignores other potentially important factors and complex regulatory networks. Given that LLPS plays a key role in multiple stages of tumorigenesis and progression, an in-depth understanding of its regulatory mechanisms and signaling pathways is essential for the development of new tumor therapeutic strategies. Therefore, LLPS shows great potential for application in tumor therapy and is expected to become an important direction for future tumor research and treatment.

First, LLPS has advantages in fighting drug-resistant tumors. Conventional treatment leads to rapid development of drug resistance in spread prostate cancer, resulting in its recurrence as castration-resistant prostate cancer (CRPC), which has a poorer prognosis and higher mortality rate, directly threatening the patient’s life. The small molecule compound ET516 inhibits the formation of LLPS of wild-type and drug-resistant mutant androgen receptor (AR), which in turn inhibits the transcriptional activity of AR, and ultimately suppresses the tumor growth of prostate cancer cells expressing drug-resistant mutant AR *in vivo (* 182). Second, constructing LLPS prognostic models to assess prognostic risk is one of the means to develop individualized treatment plans. A large number of LLPS prognostic models have the potential to characterize pathological features, cancer hallmarks, tumor microenvironment, treatment prognosis, and other information, and this conclusion has been validated in studies of LGG, ccRCC, digestive system tumors, HCC, epithelial ovarian cancer, breast cancer, endometrial cancer, BLCA, and SKCM (139, 167, 169, 170, 173–178), which provides a basis for individualized LLPS-based tumor therapy based on LLPS. Finally, LLPS technology is beneficial to antitumor drug development. Biological protein preparations such as monoclonal antibodies require high drug delivery concentration (>100 mg/ml), however, the traditional concentration methods are complex and unstable drug properties, which bring great difficulties to drug development and application. It is worth mentioning that the LLPS technology adopted by Bramham et al. has targeted solving these problems, and successfully concentrated monoclonal antibodies with high concentration (>170mg/ml) through simpler operation, and significantly improved the stability of the product, which provides new possibilities for improving the effect of tumor therapy (183).

However, while current research on LLPS is extensively pursued in the field of tumor biology, its precise translation into therapeutic applications remains bottlenecked by unresolved mechanistic complexities. The core challenges stem from dual limitations in modeling approaches and research methodologies: Model-wise, intracellular LLPS relies on multicomponent synergy (e.g., proteins, RNAs, metabolites) and subcellular compartmentalization with local concentration gradients, yet ex vivo models often oversimplify systems to single proteins or peptides, resulting in artificially elevated critical concentrations and a lack of spatial constraints (e.g., membrane structures, cytoskeletal organization) and dynamic regulatory mimicry (e.g., oxidative stress, pH fluctuations). For instance, peptide-based models developed by Leshem et al. (7)struggle to recapitulate intracellular behavior due to single-component and high critical concentration requirements. Technically, mainstream tools like confocal fluorescence microscopy and fluorescence recovery after photobleaching face hurdles in resolving three-dimensional subcompartmental environments and deciphering complex datasets (1, 184), obscuring direct links between LLPS and pathological processes such as tumorigenesis or therapeutic resistance. These combined shortcomings ultimately impede the development of precision therapeutic strategies grounded in LLPS mechanisms.

Future research should prioritize advancing scientific exploration and clinical translation of LLPS from the following perspectives: First, there is a critical need to develop *in vitro* models that better recapitulate the intracellular complexity, such as incorporating key components like RNA, metabolites, or cell extracts, while employing gene editing techniques to tag or modulate pathological mutant proteins for mechanism validation. Second, interdisciplinary technological integration must be strengthened by combining advanced instruments—including solution- and solid-state NMR techniques (185, 186), magic-angle spinning solid-state NMR (185, 187), super-resolution microscopy, electron microscopy, and single-molecule Förster resonance energy transfer—to address imaging ambiguity and data analysis challenges (188–190). Concurrently, live-cell imaging should be leveraged to directly visualize physiological LLPS dynamics, complemented by multi-omics data integration to predict intracellular phase separation behaviors, thereby enhancing research efficiency. Additionally, multidimensional experimental validation using clinical samples should be expanded to complement existing animal models and cellular assays, systematically elucidating LLPS-associated mechanisms in tumorigenesis. From the perspective of clinical translation, dual strategies are essential: optimizing pharmaceutical manufacturing processes to enhance the efficacy of existing anticancer drugs, and rationally designing novel small-molecule drugs targeting LLPS biophysical properties and condensate formation mechanisms. Synergistic advancement across these research directions will provide robust theoretical foundations for precision oncology therapies based on LLPS.

## Glossary

LLPS: Liquid–liquid phase separation
IDRs: Intrinsically Disordered Regions
DDR: DNA damage response
TFs: transcription factors
Pol: polymerase
CTD: carboxy-terminal domain
circRNA: circular RNA
ATM: ataxia-telangiectasia mutated
ATR: AT and Rad3-related protein
DNA-PK: DNA-dependent protein kinase
TopBP1: topoisomerase 2-binding protein 1
FUS: Fused in sarcoma
DSB: DNA double-strand break
CPC: chromosomal passenger complex
EB: end-binding
UBDs: ubiquitin-binding domains
LCD: low-complexity domains
Fos: fusion oncoproteins
FET: FUS/EWS/TAF15
SEs: super enhancers
RTKs: receptor tyrosine kinases
SPOP: speckle-type POZ protein
MM: multiple myeloma
NHEJ: nonhomologous end-joining
HR: homologous recombination
TCR: T cell receptor
LAT: linker for activation of T cells
SOS: Son of Sevenless
cGAS-STING: cyclic GMP-AMP synthase-stimulator of interferon genes
METTL3: methyltransferase-like protein 3
AML: acute myeloid leukemia
APA: Alternative polyadenylation
UTRs: untranslated regions
KMT2D: lysine methyltransferase 2D
HB: hepatoblastoma
MEX3A: Mex-3 RNA-binding family member A
PBs: Processing bodies
SESN2: Sestrin2
IGF2BP3: insulin-like growth factor 2 mRNA binding protein 3
DAZAP1: DAZ associated protein 1
OSCC: oral squamous cell carcinoma
COX16: cytochrome-c oxidase 16
VEGF: vascular endothelial growth factor
FOXA1: forkhead box protein A1
AR: androgen receptor
NRF1: nuclear respiratory factor 1
PCa: prostate cancer
ACP-1n: Aminocyclopropenone 1n
SCC: squamous cell carcinoma
miRNAs: MicroRNAs
miRISC: miRNA-induced silencing complex
TKIs: tyrosine kinase inhibitors
NSD2: Nuclear receptor-binding SET domain protein 2
SRC-3: steroid receptor coactivator-3
BTZ: bortezomib
CRPC: castration-resistant prostate cancer
NEPC: neuroendocrine prostate cancer
SENP1: Sentrin/SUMO-specific protease 1
TCGA: The Cancer Genome Atlas
GEO: Gene Expression Omnibus
EC: endometrial cancer
LGG: low-grade glioma
HCC: hepatocellular carcinoma
ccRCC: clear-cell renal cell carcinoma
SKCM: skin cutaneous melanoma
CRC: core regulatory circuitry
IDPs: intrinsically disordered proteins
